# Anti-tumor necrosis factor therapy in children with refractory Takayasu arteritis: report of five cases

**DOI:** 10.1186/1546-0096-9-S1-P82

**Published:** 2011-09-14

**Authors:** YO Kostina, GA Lyskina, GM Rabieva, EV Uspenskaya

**Affiliations:** 1 I.M. Sechenov First Moscow State Medical University, Department of childhood diseases, Moscow, Russia

## Background

Takayasu arteritis (TA) is a well known rare form of large vessel vasculitis characterized by granulematous inflammation. There are no controlled studies of medical treatment of children with TA.

## Aim

Tumor necrosis factor (TNF) is the most important in the formation of granulomas. The granulomatous nature of the histopathologic lesion in TA led us to consider an evaluation of the therapeutic benefits of anti-TNF therapy in patients with refractory TA.

In our study five children (5 girls) aged from 10 to 17 years with widespread inflammation in the aorta and its main branches were treated with infliximab during 6 months. The mean duration of the disease was 4.5 years. All patients received oral steroids and methotrexate or cyclophosphamide. The cause for anti-TNF treatment was standart therapy tolerance and uncontrolled arterial hypertension. Patients were tested for tuderculosis by skin test and chest roentgenography. Also we excluded active systemic infections, neutropenia, thrombocytopenia and liver dysfunction.

## Methods

The efficacy of infliximab therapy was evaluated by inflammation markers – erythrocyte sedimentation rate (ESR), C-reactive protein (CRP), fibrinogen, - clinical symptoms and results of doppler ultrasound.

## Results

The success of 3 months infliximab therapy was evaluated according to the normal significances of ESP, CRP and lack of active disease symptoms, after 6 months – there were reduced of vessel wall thickness.

## Conclusions

In children with refractory TA infliximab therapy was associated with remission and facilitating dose reduction of corticosteroids and other immunosuppressive treatment. The presented experience justifies a randomized, controlled clinical trial of anti-TNF treatment of TA in children.

